# Comparison of Hospitalization Costs Associated With Human Metapneumovirus and Respiratory Syncytial Virus Infection in US Adults

**DOI:** 10.1093/ofid/ofag202

**Published:** 2026-04-02

**Authors:** Bradley K Ackerson, Emily Rayens, Lina S Sy, Hung Fu Tseng, Lei Qian, Yi Luo, Rachelle Juan, Xuan Huang, Jennifer H Ku, Punam P Modha, Radha M Bathala, Sudhir Venkatesan, Lisa Glasser, Richard McNulty, Daniel Molnar, Chengbin Wang, Jaejin An

**Affiliations:** Department of Research & Evaluation, Kaiser Permanente Southern California, Pasadena, California, USA; Department of Research & Evaluation, Kaiser Permanente Southern California, Pasadena, California, USA; Department of Research & Evaluation, Kaiser Permanente Southern California, Pasadena, California, USA; Department of Research & Evaluation, Kaiser Permanente Southern California, Pasadena, California, USA; Department of Research & Evaluation, Kaiser Permanente Southern California, Pasadena, California, USA; Department of Research & Evaluation, Kaiser Permanente Southern California, Pasadena, California, USA; Department of Research & Evaluation, Kaiser Permanente Southern California, Pasadena, California, USA; Department of Research & Evaluation, Kaiser Permanente Southern California, Pasadena, California, USA; Center for Integrated Health Care Research, Kaiser Permanente, Honolulu, Hawaii, USA; Department of Research & Evaluation, Kaiser Permanente Southern California, Pasadena, California, USA; Department of Research & Evaluation, Kaiser Permanente Southern California, Pasadena, California, USA; BPM Evidence Statistics, BioPharmaceuticals Medical, AstraZeneca, Cambridge, UK; Vaccines and Immune Therapies, AstraZeneca, Wilmington, Delaware, USA; Vaccines and Immune Therapies, AstraZeneca, Cambridge, UK; Vaccines and Immune Therapies, AstraZeneca, Barcelona, Spain; Vaccines and Immune Therapies, AstraZeneca, Gaithersburg, Maryland, USA; Department of Research & Evaluation, Kaiser Permanente Southern California, Pasadena, California, USA

**Keywords:** adult, cost, hospitalization, human metapneumovirus, respiratory syncytial virus

## Abstract

Hospitalization costs on human metapneumovirus (hMPV) are needed to inform healthcare priorities, including vaccine and treatment development. Among 5775 adult hospitalizations with hMPV or respiratory syncytial virus (RSV), hMPV was associated with shorter length of stay but greater frequency of pneumonia, resulting in adjusted mean costs comparable to RSV hospitalization.

Acute respiratory infections caused by human metapneumovirus (hMPV) and respiratory syncytial virus (RSV) often present similarly and can significantly impact morbidity and mortality, especially in vulnerable adult and pediatric populations. Awareness of these pathogens as important causes of severe disease in high-risk and older adults may be low particularly for hMPV [1]. Although the economic impact of influenza-, SARS-CoV-2-, and RSV-associated disease in adults has been studied extensively, there are limited data on hMPV-associated healthcare costs in adult populations. A current estimate of the cost of hospitalization of adults with hMPV or RSV may increase awareness of the impact of hMPV- and RSV-associated disease in this understudied population. Furthermore, estimating the cost of hospitalization of adults with hMPV is important to the economic analysis of vaccines and treatments for hMPV currently under development prior to their introduction. Therefore, we evaluated and compared costs for a cohort of adults ≥18 years of age.

## METHODS

### Setting

This retrospective cohort study was conducted at Kaiser Permanente Southern California (KPSC), an integrated system providing healthcare at 16 large, community-based hospitals and associated clinics for >4.8 million members whose demographics closely mirrors that of the Southern California population in the United States [2]. Kaiser Permanente Southern California electronic health records include sociodemographics, healthcare utilization, diagnoses, laboratory tests, procedures, pharmacy utilization, vaccinations, and membership history.

### Study Cohort

The study cohort consisted of hospitalized adults ≥18 years of age who tested positive for hMPV or RSV by reverse transcription-polymerase chain reaction (RT-PCR) between 7 days prior to 2 days following admission between 01 July 2011 and 30 June 2024 from KPSC. Hospitalizations with <1 year of continuous KPSC membership before admission or missing diagnosis-related group (DRG) codes were excluded from analysis.

The analysis included the first hMPV or RSV test-positive hospitalization per season (1 July to 30 June) and repeat hospitalizations for the same individual in separate seasons were included. The unit of analysis was hospitalization. The KPSC Institutional Review Board approved this study and waived the requirement for informed consent.

### Outcome

The primary outcome of the study was total hospitalization costs. Each hospitalization record included a DRG code based on principal and secondary diagnoses and procedures. Using a utilization-based cost algorithm [3], we assigned nationally recognized direct healthcare costs to each hospitalization record. Daily average cost per DRG was derived from patients ≥18 years of age using the 2020 National Inpatient Sample (NIS) [4]. Total hospitalization costs were calculated by multiplying mean daily cost per DRG derived from the 2020 NIS by length of stay (LOS) for each hospital record. To compare costs across published studies and our findings, we applied the consumer price index for medical care services to report costs in 2020 US dollars (USD).

### Statistical Analysis

We described patient sociodemographic and clinical characteristics at hospital admission. Clinically relevant variables including age, sex, race/ethnicity, baseline healthcare utilization, immunocompromised status, Charlson comorbidity score, and year and month of hospital admission were included as covariates in adjusted models. Absolute standardized differences (ASDs) between hMPV and RSV hospitalizations were calculated; variables with ASD >0.1 were included as additional covariates. *t*-tests and χ^2^ tests were used to compare mean LOS, occurrence of complications during the hospital stay, and proportion of DRG codes in hMPV and RSV hospitalizations.

Generalized linear regression models (GLMs) with a gamma distribution and log link were used to estimate the population mean cost with 95% confidence intervals (CIs) for hMPV and RSV hospitalizations. Differences in mean cost with 95% CI between hMPV and RSV hospitalizations were estimated using GLMs. Model-based contrasts were reported for both unadjusted and adjusted cost estimates. Stratified analyses were conducted based on sex (male, female), age at hospital admission (18–49, 50–59, 60–74, and ≥75 years), and pre- and postappearance of COVID-19. Sensitivity analyses were conducted by including hospitalizations with missing DRGs. Costs for missing DRG records were imputed using predicted values from a GLM applied to the entire sample. Additional sensitivity analyses were conducted by selecting appropriate families and link functions based on the modified Park, Pregibon link, Pearson correlation, and modified Hosmer and Lemeshow tests [5] and by accounting for within-person correlation in repeated hospitalizations.

All statistical analyses were performed using SAS 9.4 (SAS Institute) or Stata 18 (StataCorp LLC). *P* < .05 was considered statistically significant.

## RESULTS

We included 5775 hospitalizations among 5650 adults who tested positive for hMPV (n = 2810 hospitalizations) or RSV (n = 2965 hospitalizations) in this analysis. Compared with patients with RSV, those with hMPV were younger and had lower proportions with kidney disease, Charlson comorbidity score ≥2, or immunocompromising conditions. Admissions associated with hMPV occurred more frequently between January and June, while RSV admissions were more common between October and March (ASD >0.1; Table 1).

**Table 1.** Baseline Patient Characteristics Associated With Hospitalization for hMPV and RSV

	hMPV Hospitalizations N = 2810^a^	RSV Hospitalizations N = 2965^b^	ASD
Age on date of admission, year	…	…	0.103
Mean (SD)	71.0 (16.1)	72.6 (15.4)	…
Median (Q1, Q3)	74 (62.0, 83.0)	75 (64.0, 84.0)	…
Age on date of admission, year, n (%)	…	…	0.105
18–49	299 (10.6)	244 (8.2)	…
50–59	307 (10.9)	286 (9.6)	…
60–74	861 (30.6)	900 (30.4)	…
&ge75	1343 (47.8)	1535 (51.8)	…
Sex, n (%)	…	…	0.032
Female	1673 (59.5)	1718 (57.9)	…
Male	1137 (40.5)	1247 (42.1)	…
Race/ethnicity, n (%)	…	…	0.064
Non-Hispanic White	1295 (46.1)	1343 (45.3)	…
Non-Hispanic Black	309 (11.0)	374 (12.6)	…
Hispanic	843 (30.0)	906 (30.6)	…
Non-Hispanic Asian	315 (11.2)	296 (10.0)	…
Other/multiple/unknown	48 (1.7)	46 (1.6)	…
Length of continuous membership,^c^ year, n (%)	…	…	0.067
1–5	543 (19.3)	522 (17.6)	…
6–10	435 (15.5)	416 (14.0)	…
≥11	1832 (65.2)	2027 (68.4)	…
Number of prior outpatient and virtual visits,^d^ n (%)	…	0.054	…
0	50 (1.8)	42 (1.4)	…
1–4	338 (12.0)	346 (11.7)	…
5–10	705 (25.1)	696 (23.5)	…
≥11	1717 (61.1)	1881 (63.4)	…
Number of prior emergency department visits,^d^ n (%)	…	0.055	…
0	1146 (40.8)	1134 (38.2)	…
1	692 (24.6)	742 (25.0)	…
≥2	972 (34.6)	1089 (36.7)	…
Number of prior hospitalizations,^d^ n (%)	…	…	0.096
0	1788 (63.6)	1761 (59.4)	…
1	535 (19.0)	591 (19.9)	…
≥2	487 (17.3)	613 (20.7)	…
Vaccination,^d^ n (%)	…	…	…
Influenza vaccine	1946 (69.3)	2063 (69.6)	0.007
COVID-19 vaccine	401 (14.3)	384 (13.0)	0.039
RSV vaccine	28 (1.0)	6 (0.2)	0.103
Chronic diseases,^d^ n (%)	…	…	…
Kidney disease	1116 (39.7)	1327 (44.8)	0.102
Heart disease	1079 (38.4)	1194 (40.3)	0.038
Lung disease	1374 (48.9)	1506 (50.8)	0.038
Liver disease	260 (9.3)	278 (9.4)	0.004
Diabetes	1298 (46.2)	1407 (47.5)	0.025
Immunocompromised on date of admission, n (%)	536 (19.1)	688 (23.2)	0.101
Charlson comorbidity score^d,e^	…	…	0.069
Mean (SD)	4.5 (2.6)	4.7 (2.7)	…
Median (Q1, Q3)	4 (2.0, 6.0)	4 (2.0, 6.0)	…
Charlson comorbidity score,^d,e^ n (%)	…	…	0.132
0	261 (9.3)	191 (6.4)	…
1	342 (12.2)	299 (10.1)	…
≥2	2207 (78.5)	2475 (83.5)	…
Frailty index^d,f^	…	…	0.074
Mean (SD)	0.19 (0.06)	0.19 (0.06)	…
Median (Q1, Q3)	0.18 (0.15, 0.22)	0.19 (0.15, 0.23)	…
Frailty index,^d,f^ n (%)	…	…	0.073
Q1 (0.15), least frail	746 (26.5)	700 (23.6)	…
Q2 (0.18)	700 (24.9)	745 (25.1)	…
Q3 (0.23)	691 (24.6)	750 (25.3)	…
Q4 (0.43), most frail	673 (24.0)	770 (26.0)	…
Body mass index,^g^ kg/m^2^, n (%)	…	…	0.079
<18.5	112 (4.0)	125 (4.2)	…
18.5 to <25	739 (26.3)	827 (27.9)	…
25 to <30	752 (26.8)	848 (28.6)	…
30 to <40	840 (29.9)	832 (28.1)	…
≥40	342 (12.2)	310 (10.5)	…
Unknown	25 (0.9)	23 (0.8)	…
Smoking,^g^ n (%)	…	…	0.066
Never	1692 (60.2)	1690 (57.0)	…
Ever	1096 (39.0)	1252 (42.2)	…
Unknown	22 (0.8)	23 (0.8)	…
Medicaid, n (%)	372 (13.2)	371 (12.5)	0.022
Neighborhood median household income, n (%)	…	…	0.043
<$40 000	258 (9.2)	301 (10.2)	…
$40 000–$59 999	714 (25.4)	728 (24.6)	…
$60 000–$79 999	675 (24.0)	736 (24.8)	…
≥$80 000	1160 (41.3)	1196 (40.3)	…
Unknown	3 (0.1)	4 (0.1)	…
ARI/LRTD on date of admission, n (%)	2709 (96.4)	2837 (95.7)	0.037
Year of seasons (July to June), n (%)	…	…	0.454
2011–2012	55 (2.0)	23 (0.8)	…
2012–2013	27 (1.0)	135 (4.6)	…
2013–2014	244 (8.7)	122 (4.1)	…
2014–2015	150 (5.3)	312 (10.5)	…
2015–2016	404 (14.4)	315 (10.6)	…
2016–2017	277 (9.9)	460 (15.5)	…
2017–2018	351 (12.5)	386 (13.0)	…
2018–2019	346 (12.3)	256 (8.6)	…
2019–2020	171 (6.1)	200 (6.7)	…
2020–2021	0 (0.0)	4 (0.1)	…
2021–2022	75 (2.7)	84 (2.8)	…
2022–2023	371 (13.2)	263 (8.9)	…
2023–2024	339 (12.1)	405 (13.7)	…
Month of hospital admission, n (%)	…	…	0.757
July to September	96 (3.4)	33 (1.1)	…
October to December	264 (9.4)	752 (25.4)	…
January to March	1657 (59.0)	2026 (68.3)	…
April to June	793 (28.2)	154 (5.2)	…
Coinfection,^h^ n (%)	…	…	…
Adenovirus	7 (0.2)	8 (0.3)	0.004
Coronavirus (229E, HKU1, NL63, OC43)	28 (1.0)	37 (1.2)	0.024
SARS-CoV-2	5 (0.2)	9 (0.3)	0.026
hMPV	NA	11 (0.4)	NA
Human rhinovirus/enterovirus	48 (1.7)	79 (2.7)	0.065
Influenza	16 (0.6)	44 (1.5)	0.091
Parainfluenza	8 (0.3)	9 (0.3)	0.004
RSV	11 (0.4)	NA	NA
*Chlamydia pneumoniae*	0	0	NA
*Mycoplasma pneumoniae*	0	0	NA

Abbreviations: ARI, acute respiratory infection; ASD, absolute standardized difference; hMPV, human metapneumovirus; LRTD, lower respiratory tract disease; n, number; NA, not applicable; Q, quartile; RSV, respiratory syncytial virus; SD, standard deviation.

^a^Representing 2800 individuals.

^b^Representing 2949 individuals.

^c^Defined based on all available medical records prior to the hospital admission date.

^d^Defined in the 1 year prior to the hospital admission date.

^e^Possible range: 0–29. A weighted score of major comorbidities, assigning higher weights to severe conditions, which predicts mortality. Quan H, et al. Updating and validating the Charlson comorbidity index and score for risk adjustment in hospital discharge abstracts using data from 6 countries. *American Journal of Epidemiology* 173.6 (2011): 676–682.

^f^Possible range: 0–1. A validated claims-based frailty index which predicts disability, falls, and skilled nursing facility use. Kim DH, et al. Measuring frailty in Medicare data: development and validation of a claims-based frailty index. *The Journals of Gerontology: Series A* 73.7 (2018): 980–987.

^g^Defined as most recent assessment in 2 years prior to and including the hospital admission date.

^h^Limited to respiratory pathogen panel tests, descriptive only; some pathogen results may not be available for the entire study period.

The most frequent DRG codes were septicemia with multiple chronic conditions (∼30%) and pneumonia with multiple chronic conditions (∼10%) in both groups (Supplementary Table 1). Hospitalization with hMPV had slightly shorter mean LOS (6.4 vs 6.8 days, *P* = .018) but similar proportions with intensive care unit (ICU) admission (14.8% vs 16.5%, *P* = .084) and respiratory support requirement (24.8% vs 25.7%, *P* = .434) and a greater proportion with pneumonia (66.7% vs 56.8%, *P* <.001) when compared with hospitalization with RSV (Supplementary Table 2).

Adjusted mean hospitalization costs were $20 188 (95% CI: $19 317, $21 060) for hMPV and $21 759 (95% CI: $20 847, $22 670) for RSV, with a difference of −$1066 (95% CI: −$2413, $281; Table 2). Adjusted mean differences stratified by sex, age, and time period relative to the COVID-19 pandemic ranged from −$2371 (95% CI: −$6739, $1996) to $1521 (95% CI: −$2606, $5647). Sensitivity analysis including hospitalizations with missing DRG codes, as well as using alternative families and link functions and accounting for within-person correlation, showed consistent results (Supplementary Table 3).

**Table 2.** Comparison of Crude and Adjusted Costs in 2020 US Dollars Between hMPV and RSV Hospitalizations

	HMPV Hospitalizations			RSV Hospitalizations			Difference In Mean Cost (hMPV−RSV) (95% CI)	
Outcome	N	Crude Mean Cost (SD)	Adjusted Mean Cost^a^ (95% CI)	N	Crude Mean Cost (SD)	Adjusted Mean Cost^a^ (95% CI)	Unadjusted^b^	Adjusted^a,c^
Overall	2810	20 102 (22 011)	20 188 (19 317, 21 060)	2965	21 852 (29 816)	21 759 (20 847, 22 670)	–1753 (–3101, –405)	–1066 (–2413, 281)
Sex								
Male	1137	20 232 (21 064)	20 493 (19 079, 21 907)	1247	21 772 (29 468)	21 425 (20 024, 22 826)	–1540 (–3633, 553)	–936 (–2955, 1084)
Female	1673	20 014 (22 637)	20 407 (19 234, 21 580)	1718	21 910 (30 075)	21 565 (20 357, 22 772)	–1896 (–3648, –144)	–1155 (–2862, 552)
Age on date of admission, y								
18–49	299	20 911 (25 702)	21 968 (18 985, 24 951)	244	21 313 (25 797)	20 431 (17 389, 23 473)	–401 (–4716, 3914)	1521 (–2606, 5647)
50–59	307	21 793 (26 266)	21 763 (18 918, 24 608)	286	24 996 (51 095)	24 101 (20 838, 27 364)	–3203 (–7775, 1369)	–2371 (–6739, 1996)
60–74	861	20 127 (21 325)	20 457 (18 852, 22 063)	900	23 345 (34 083)	22 662 (20 932, 24 392)	–3218 (–5674, –763)	–2221 (–4618, 176)
≥75	1343	19 520 (20 432)	19 826 (18 545, 21 107)	1535	20 476 (21 010)	20 465 (19 244, 21 687)	–957 (–2723, 810)	–636 (–2398, 1125)
Periods^d^								
Preappearance of COVID-19	2025	20 277 (22 290)	20 487 (19 424, 21 550)	2209	21 876 (30 884)	21 439 (20 372, 22 506)	–1598 (–3173, –23)	–961 (–2485, 562)
Postappearance of COVID-19	785	19 651 (21 281)	20 311 (18 605, 22 017)	756	21 784 (26 468)	21 719 (19 745, 23 692)	–2133 (–4706, 440)	–1391 (–3990, 1208)

Abbreviations: CI, confidence interval; GLM, generalized linear model; hMPV, human metapneumovirus; N, number; RSV, respiratory syncytial virus; SD, standard deviation; US, United States.

^a^Population-averaged predictions from a GLM with a gamma distribution and log link, adjusted for age, sex, race/ethnicity, baseline healthcare utilization, kidney disease, immunocompromised status, Charlson comorbidity score, and year and month of hospitalization.

^b^Unadjusted mean differences are model-based contrasts from a GLM, representing the average cost difference between hMPV and RSV hospitalizations without covariate adjustment.

^c^Adjusted mean differences are model-based contrasts from a GLM, representing the average cost difference between hMPV and RSV hospitalizations after accounting for all covariates.

^d^Preappearance of COVID-19 period is defined from 1 July 2011 to 30 June 2020; postappearance of COVID-19 period is defined from 1 July 2020 to 30 June 2024.

## DISCUSSION

This study indicates that hospitalization of adults with hMPV or RSV is associated with comparable and substantial healthcare costs. To our knowledge, this study is the first to describe the healthcare costs of a large cohort of adults hospitalized with laboratory-confirmed hMPV. All infections were detected by multiplex RT-PCR, which allows simultaneous detection of hMPV and RSV, among other respiratory pathogens. Therefore, testing conducted for clinical reasons, often to validate a diagnosis of influenza or SARS-CoV-2, frequently revealed hMPV or RSV infection that would have otherwise gone undetected. Respiratory pathogen testing was performed within 2 days following hospital admission among >97% of hospitalizations, reducing the likelihood that nosocomial infections impacted the analysis substantially. Furthermore, nearly 50% of tests were obtained at least 1 day after admission and test results were often not immediately available, so that initial clinical management decisions and their associated costs were unlikely to be influenced substantially by virologic diagnosis.

Despite growing evidence that hMPV and RSV infections are associated with severe illness in older adults and adults with comorbid conditions, there are limited data on the cost of hMPV-associated hospitalizations in adults. However, several studies have estimated the cost of hospitalization for RSV. A recent study estimated that RSV disease among US adults ≥60 years of age resulted in nearly 8.2 million outpatient visits, 826 790 hospitalizations, and 65 853 deaths over 5 years, resulting in nearly $3 billion in direct medical costs annually without RSV vaccination [6]. In a study spanning 15 seasons between 1997 and 2012, the cost of RSV-associated hospitalization of adults was found to be much higher than our estimate when adjusted to 2020 USD ($45 880) [7]. However, this analysis included hospitalizations prior to 2008, at which time more sensitive molecular testing for RSV became available. Therefore, their analysis may have included patients with RSV disease that was more severe than RSV disease detected by more sensitive methods in our study. Similarly, a recent study estimated the cost of hospitalization for RSV-associated lower respiratory tract infection to be $37 025 in 2023 USD, which is higher than our estimate even when adjusted to 2020 USD ($34 975) [8]. However, their estimate included claims for professional service fees that are not included in our DRG-based estimated cost. We previously found that the cost of hospitalization of older adults between 2011 and 2015 for RSV was at least as high as that for influenza ($16 034 vs $15 163, respectively, in 2013 USD), and the RSV cost adjusted for inflation ($198 71 2020 USD) [9] was similar to cost estimates in this study for hospitalization with hMPV or RSV. Although these hospitalization cost estimates may help inform cost-effectiveness analyses as vaccine and therapeutics directed against hMPV and RSV are developed, such analyses will require country-specific inputs.

The burden of disease associated with RSV and with hMPV is considerable. A landmark study of high-risk adults and adults ≥65 years of age in the United States detected RSV infection in 3%–7% of healthy community-dwelling older adults, 4%–10% of high-risk adults, and 16% of adults hospitalized with cardiopulmonary conditions [10]. In addition, RSV infection was estimated to result in ∼177 000 hospitalizations and 10 000–14 000 deaths [10]. Similarly, hMPV was estimated recently to cause over 545 000 infections and 122 000 hospitalizations among older adults in the United States, suggesting that the burden of hMPV-associated disease is comparable to that of RSV-associated disease [11, 12], although the clinical impact of hMPV and RSV may vary between seasons [13]. Severity of disease among adults hospitalized with hMPV or RSV is also similar. A recent study found that the rates of detection of hMPV and RSV among hospitalized adults ≥18 years of age in whom respiratory viruses were detected were similar (8.1% vs 9.8%, respectively). Furthermore, the LOS (4.8 vs 4.4 days) and proportion admitted to the ICU (16.5% vs 16.1%) were also comparable [14]. Likewise, a multicenter European study that compared the clinical burden of hMPV, parainfluenza virus, and RSV in hospitalized adults found that infection with RSV or hMPV results in similar hospital and ICU admission rates and morbidity and mortality among hospitalized adults [15].

Our analysis of hMPV- and RSV-associated hospitalization costs should be interpreted with caution. Since we assigned nationally representative costs to healthcare utilization, these costs may be more generalizable but may not be the actual costs for KPSC. Furthermore, NIS does not include professional fees, which may underestimate the overall healthcare cost of hospitalized adults. Additionally, the cost estimates in this study may not be generalizable to other settings and testing for hMPV and RSV was likely incomplete due to under recognition of their ability to cause serious disease. However, the comparison of the cost of hospitalization of adults with hMPV versus RSV should not be affected substantially as these limitations impact both the estimated costs of hMPV- and RSV-associated hospitalization [9]. Finally, in our results, the 95% CI of cost difference between hMPV- and RSV-associated hospitalizations crosses zero, so findings reflect no statistically significant differences rather than formal equivalence.

## CONCLUSION

Both hMPV and RSV are important causes of severe illness in hospitalized adults that are associated with high hospitalization costs that are not statistically significantly different. The increasing proportion of older adults combined with the high cost of hospitalization for hMPV and RSV underscore the importance of the development of vaccines and therapeutics directed against both hMPV and RSV. Increased provider awareness of the burden and high cost of hMPV and RSV disease will be important to the utilization of these interventions when they become available.

## Supplementary Material

ofag202_Supplementary_Data

## Supplementary Data

Supplementary materials are available at *Open Forum Infectious Diseases* online. Consisting of data provided by the authors to benefit the reader, the posted materials are not copyedited and are the sole responsibility of the authors, so questions or comments should be addressed to the corresponding author.
