# Supplementary material for: Comparison of Hospitalization Costs Associated With Human Metapneumovirus and Respiratory Syncytial Virus Infection in US Adults
Source: Open Forum Infect Dis. 2026 Apr 2;13(4):ofag202. doi: 10.1093/ofid/ofag202 (PMC13095376; doi:10.1093/ofid/ofag202)
Supplement: ofag202_Supplementary_Data [file ofag202_supplementary_data.docx]

Comparison of Hospitalization Costs Associated with Human Metapneumovirus and Respiratory Syncytial Virus Infection in US Adults

**Authors:**

Bradley K. Ackerson^1^, Emily Rayens^1^, Lina S. Sy^1^, Hung Fu Tseng^1^, Lei Qian^1^, Yi Luo^1^, Rachelle Juan^1^, Xuan Huang^1^, Jennifer H. Ku^2^, Punam P. Modha^1^, Radha M. Bathala^1^, Sudhir Venkatesan^3^, Lisa Glasser^4^, Richard McNulty^5^, Daniel Molnar^6^, Chengbin Wang^7^, Jaejin An^1^

**Affiliations:**

^1^Department of Research & Evaluation, Kaiser Permanente Southern California, Pasadena, CA, US

^2^Center for Integrated Health Care Research, Kaiser Permanente, Honolulu, HI, US

^3^BPM Evidence Statistics, BioPharmaceuticals Medical, AstraZeneca, Cambridge, UK

^4^Vaccines & Immune Therapies, AstraZeneca, Wilmington, DE, US

^5^Vaccines & Immune Therapies, AstraZeneca, Cambridge, UK

^6^Vaccines & Immune Therapies, AstraZeneca, Barcelona, Spain

^7^Vaccines & Immune Therapies, AstraZeneca, Gaithersburg, MD, US

**Corresponding Author:**

Bradley K. Ackerson, M.D.

Research & Evaluation, Southern California Permanente Medical Group

100 S. Los Robles Avenue, 2^nd^ Floor

Pasadena, CA 91101

United States of America

Tel: (310) 702-5141

Email: Bradley.K.Ackerson@kp.org

**Alternate Corresponding Author:**

Jaejin An, Ph.D.

Department of Research & Evaluation, Southern California Permanente Medical Group

100 S. Los Robles Avenue, 2^nd^ Floor

Pasadena, CA 91101

United States of America

Tel: 626-564-5906

Email: Jajin.X.An@kp.org

**Supplemental Data**

1. Supplemental Table 1. Frequency of 20 most common DRGs for hMPV and RSV hospitalizations
2. Supplemental Table 2. Length of stay and complications for hMPV and RSV hospitalizations
3. Supplemental Table 3. Comparison of crude and adjusted costs in 2020 US dollars between hMPV and RSV hospitalizations, sensitivity analysis after imputing cost for missing DRGs and changing the family, link function, and accounting for within-person correlation

**Supplemental Table 1. Frequency of 20 most common DRGs for hMPV and RSV hospitalizations**

| **DRG** | **hMPV hospitalizations (N=2,810)** | **RSV**  **hospitalizations (N=2,965)** | **p-value** |
| --- | --- | --- | --- |
| 871 SEPTICEMIA OR SEVERE SEPSIS WITHOUT MV >96 HOURS WITH MCC | 887 (31.6%) | 818 (27.6%) | 0.002 |
| 193 SIMPLE PNEUMONIA AND PLEURISY WITH MCC | 303 (10.8%) | 269 (9.1%) | 0.042 |
| 189 PULMONARY EDEMA AND RESPIRATORY FAILURE | 189 (6.7%) | 243 (8.2%) | 0.023 |
| 194 SIMPLE PNEUMONIA & PLEURISY W CC | 211 (7.5%) | 173 (5.8%) | 0.015 |
| 202 BRONCHITIS AND ASTHMA WITH CC/MCC | 145 (5.2%) | 198 (6.7%) | 0.010 |
| 190 CHRONIC OBSTRUCTIVE PULMONARY DISEASE WITH MCC | 141 (5.0%) | 147 (5.0%) | 0.995 |
| 291 HEART FAILURE AND SHOCK WITH MCC | 120 (4.3%) | 157 (5.3%) | 0.053 |
| 191 CHRONIC OBSTRUCTIVE PULMONARY DISEASE WITH CC | 68 (2.4%) | 86 (2.9%) | 0.224 |
| 872 SEPTICEMIA OR SEVERE SEPSIS W/O MV 96+ HOURS W/O MCC | 54 (1.9%) | 82 (2.8%) | 0.028 |
| 870 SEPTICEMIA OR SEVERE SEPSIS W MV 96+ HOURS | 37 (1.3%) | 47 (1.6%) | 0.362 |
| 208 RESPIRATORY SYSTEM DIAGNOSIS W VENTILATOR SUPPORT <96 HOURS | 25 (0.9%) | 48 (1.6%) | 0.011 |
| 203 BRONCHITIS & ASTHMA W/O CC/MCC | 30 (1.1%) | 40 (1.3%) | 0.302 |
| 153 OTITIS MEDIA AND URI WITHOUT MCC | 28 (1.0%) | 39 (1.3%) | 0.236 |
| 195 SIMPLE PNEUMONIA AND PLEURISY WITHOUT CC/MCC | 40 (1.4%) | 26 (0.9%) | 0.057 |
| 280 ACUTE MYOCARDIAL INFARCTION, DISCHARGED ALIVE WITH MCC | 27 (1.0%) | 37 (1.2%) | 0.274 |
| 177 RESPIRATORY INFECTIONS & INFLAMMATIONS W MCC | 33 (1.2%) | 21 (0.7%) | 0.073 |
| 853 INFECTIOUS & PARASITIC DISEASES W O.R. PROCEDURE W MCC | 22 (0.8%) | 26 (0.9%) | 0.662 |
| 207 RESPIRATORY SYSTEM DIAGNOSIS W VENTILATOR SUPPORT 96+ HOURS | 17 (0.6%) | 27 (0.9%) | 0.168 |
| 152 OTITIS MEDIA & URI W MCC | 23 (0.8%) | 15 (0.5%) | 0.153 |
| 308 CARDIAC ARRHYTHMIA & CONDUCTION DISORDERS W MCC | 18 (0.6%) | 20 (0.7%) | 0.842 |

CC = complication or comorbidity, DRG = diagnosis related group, hMPV = human metapneumovirus, MCC = major complication or comorbidity, MV = mechanical ventilation, O.R. = operating room, RSV = respiratory syncytial virus, URI = upper respiratory infection, W = with, W/O = without

**Supplemental Table 2. Length of stay and complications for hMPV and RSV hospitalizations**

| **Outcomes** | **hMPV hospitalizations (N=2,810)** | **RSV**  **hospitalizations (N=2,965)** | **p-value** |
| --- | --- | --- | --- |
| Length of hospital stay, days |  |  |  |
| Mean (SD) | 6.4 (5.7) | 6.8 (6.4) | 0.018 |
| Median (Q1, Q3) | 5 (4.0, 7.0) | 5 (4.0, 7.0) |  |
| ICU length of stay, days |  |  |  |
| Mean (SD) | 6.4 (6.5) | 6.6 (7.6) | 0.781 |
| Median (Q1, Q3) | 4 (2.0, 8.0) | 4 (2.0, 7.0) |  |
| Length of hospital stay ≥7 days, n (%) | 853 (30.4) | 1,005 (33.9) | 0.004 |
| Use of respiratory support^*^, n (%) | 697 (24.8) | 762 (25.7) | 0.434 |
| ICU admission, n (%) | 417 (14.8) | 489 (16.5) | 0.084 |
| Occurrence of complications, n (%) |  |  |  |
| Pneumonia | 1,875 (66.7) | 1,685 (56.8) | <0.001 |
| Bacteremia, sepsis | 1,088 (38.7) | 1,108 (37.4) | 0.291 |
| Septic shock | 120 (4.3) | 153 (5.2) | 0.111 |
| Cardiac complications | 702 (25.0) | 808 (27.3) | 0.050 |
| Neurovascular complications | 75 (2.7) | 80 (2.7) | 0.946 |
| Acute renal failure | 610 (21.7) | 682 (23.0) | 0.238 |
| Exacerbation of chronic conditions, n (%) |  |  |  |
| Asthma | 408 (14.5) | 418 (14.1) | 0.647 |
| COPD | 545 (19.4) | 610 (20.6) | 0.263 |
| Bronchiectasis | 23 (0.8) | 24 (0.8) | 0.969 |
| In-hospital death, n (%) | 101 (3.6) | 120 (4.0) | 0.370 |

^*^Receipt of invasive mechanical ventilation, non-invasive positive pressure ventilation and/or high-flow nasal cannula oxygen. P-values were calculated using t-tests for continuous variables and chi-square tests for categorical variables.

COPD = chronic obstructive pulmonary disease, hMPV= human metapneumovirus, ICU = intensive care unit, N, n = number, Q = quartile, RSV= respiratory syncytial virus, SD = standard deviation

**Supplemental Table 3. Comparison of crude and adjusted costs in 2020 US dollars between hMPV and RSV hospitalizations, sensitivity analysis after imputing cost for missing DRGs and changing the family, link function, and accounting for within-person correlation**

|  | **hMPV hospitalizations** | | | | **RSV hospitalizations** | | | | **Difference in Mean Cost**  **(hMPV-RSV) (95% CI)** | |
| --- | --- | --- | --- | --- | --- | --- | --- | --- | --- | --- |
| **Sensitivity Analyses** | **N** | **Crude Mean Cost**  **(SD)** | **Adjusted Mean**  **Cost^a^**  **(95% CI)** | **N** | | **Crude Mean Cost**  **(SD)** | **Adjusted Mean**  **Cost^a^**  **(95% CI)** | **Unadjusted^b^** | | **Adjusted**^a,c^ |
| After imputing cost for missing DRGs^d^ | 2,859 | 19,857  (21,904) | 19,945  (19,085, 20,804) | 3,029 | | 21,524  (29,590) | 21,429  (20,535, 22,324) | –1,670  (–2,997, –344) | | –993  (–2,319, 333) |
| After changing the family and link function^e^ | 2,810 | 20,102  (22,011) | 20,188  (19,317, 21,060) | 2,965 | | 21,852  (29,816) | 21,759  (20,847, 22,670) | –1,766  (–3,137, –395) | | –938  (–2,278, 401) |
| After accounting for within-person correlation | 2,810 | 20,102  (22,011) | 20,188  (19,380, 20,997) | 2,965 | | 21,852  (29,816) | 21,759  (20,774, 22,744) | -1,753  (-3,103, -402) | | -1,066  (-2,312, 180) |

^a^ Population-averaged predictions from a generalized linear model with a gamma distribution and log link, adjusted for age, sex, race/ethnicity, baseline healthcare utilization, kidney disease, immunocompromised status, Charlson comorbidity score, and year and month of hospitalization.

^b^ Unadjusted mean differences are model-based contrasts from a generalized linear model, representing the average cost difference between hMPV and RSV hospitalizations without covariate adjustment.

^c^ Adjusted mean differences are model-based contrasts from a generalized linear model, representing the average cost difference between hMPV and RSV hospitalizations after accounting for all covariates.

^d^ Cost imputed for hospital records with missing DRG codes using generalized linear model.

^e^ Sensitivity analysis using a generalized linear model with an inverse Gaussian distribution and a data-driven optimal power link.

CI = confidence interval, DRG = diagnosis related group, hMPV = human metapneumovirus, N = number, RSV = respiratory syncytial virus, SD = standard deviation, US = United States
